# Ultrasound-based intratumoral and peritumoral radiomics for preoperative prediction of lymph node metastasis in pancreatic ductal adenocarcinoma

**DOI:** 10.3389/fmed.2026.1793048

**Published:** 2026-08-19

**Authors:** Yanhua Huang, Hongwei Qian, Li Bao, Hong Fu, Jianhua Yu, Baochun Lu, Zhihong Shen

**Affiliations:** 1 Department of Ultrasound, Shaoxing People's Hospital, Shaoxing, China; 2 School of Medicine, Shaoxing University, Shaoxing, Zhejiang, China; 3 Department of Hepatobiliary and Pancreatic Surgery, Shaoxing People's Hospital, Shaoxing, China; 4 Shaoxing Key Laboratory of Minimally Invasive Abdominal Surgery and Precise Treatment of Tumor, Shaoxing, China

**Keywords:** lymph node metastasis, pancreatic ductal adenocarcinoma, peritumoral region, radiomics, ultrasound

## Abstract

**Background:**

Accurate preoperative assessment of lymph node metastasis (LNM) in pancreatic ductal adenocarcinoma (PDAC) remains challenging. We developed and compared ultrasound-based intratumoral, peritumoral, clinical, and combined models for LNM prediction and explored the complementary value of multi-regional imaging.

**Methods:**

Ninety-nine patients with pathologically confirmed PDAC who underwent preoperative ultrasound were retrospectively enrolled. Intratumoral and 3-mm peritumoral ROIs were manually delineated. Radiomics features were extracted using PyRadiomics and selected via reproducibility filtering, correlation analysis, and LASSO. Nine machine-learning algorithms were evaluated to identify the optimal classifier for each region (intratumoral: logistic regression; peritumoral: random forest). A decision-level combined model was constructed by integrating regional model outputs with clinical information. Discrimination was assessed by AUC with sensitivity, specificity, accuracy, PPV, and NPV; calibration by calibration curves and the Hosmer–Lemeshow test; clinical utility by decision curve analysis (DCA). Model interpretability and inter-model relationships were explored using SHAP, correlation, and Bland–Altman analyses.

**Results:**

The intratumoral and peritumoral models achieved AUCs of 0.815 and 0.792, respectively. The combined model yielded the highest performance (AUC = 0.898, 95% CI: 0.770–1.000) with good calibration (Hosmer–Lemeshow *p* = 0.091) and the greatest net benefit on DCA. DeLong tests showed no statistically significant AUC differences among models. Intratumoral and peritumoral outputs were moderately correlated (Pearson’s *r* = 0.711, *p* < 0.001), and Bland–Altman analysis demonstrated overall agreement with several outliers, suggesting complementary region-specific information.

**Conclusion:**

Intratumoral and peritumoral ultrasound radiomics provide complementary information for preoperative LNM prediction in PDAC. The decision-level combined model achieved numerically higher discrimination with favorable calibration, supporting further validation before clinical translation.

## 1 Introduction

Pancreatic ductal adenocarcinoma (PDAC) is a highly aggressive malignancy with a dismal prognosis, characterized by a five-year survival rate of less than 10% (1, 2). Because of the asymptomatic nature of early-stage disease, many patients are diagnosed at advanced stages, often with lymph node or distant metastases (3). Accurate preoperative assessment of lymph node metastasis (LNM) is therefore essential for staging, treatment planning, and improving patient outcomes (4).

Pathological examination remains the gold standard for determining nodal status (5), but this approach is inherently postoperative, causing delays and providing no guidance for preoperative management (6). Conventional imaging techniques such as contrast-enhanced CT, MRI, and PET are widely used, yet they often fail to differentiate metastatic lymph nodes from those enlarged due to benign causes (7, 8). Moreover, diagnostic performance is subject to interobserver variability among radiologists (9, 10).

Radiomics, which enables extraction of high-throughput quantitative features from medical images, has emerged as a promising approach for capturing tumor heterogeneity beyond conventional assessment (11). It has been increasingly applied in oncology, with reported success in breast, lung, and liver cancers (12). In PDAC, several CT-based radiomics studies have explored LNM prediction. For example, Chen et al. (5) developed a deep learning model based on CT images that accurately predicted nodal metastasis, while Bian et al. (13) and Fu et al. (14) demonstrated improved performance by integrating imaging and clinical features. These findings underscore the potential of radiomics for LNM assessment in PDAC.

Compared with CT and MRI, ultrasound (US) offers unique advantages such as real-time imaging, non-invasiveness, cost-effectiveness, and broad clinical availability. However, the application of ultrasound radiomics in PDAC remains limited. Furthermore, most radiomics studies to date have concentrated on intratumoral features, even though the peritumoral region—the tumor microenvironment—plays a critical role in invasion and metastatic dissemination. Recent studies in hepatocellular carcinoma have demonstrated the added predictive value of peritumoral features for microvascular invasion and recurrence (15, 16). Yet, no systematic investigations have examined the combined use of intratumoral and peritumoral features from US images for predicting LNM in PDAC.

Therefore, our study aimed to develop and compare radiomics models based on intratumoral, peritumoral, and combined features derived from preoperative US images to predict LNM in patients with PDAC. In addition, different fusion strategies were evaluated, and the potential clinical relevance and interpretability of the models were investigated.

## 2 Materials and methods

### 2.1 Study population

This retrospective study included patients who underwent curative surgical resection for pathologically confirmed PDAC at Shaoxing People’s Hospital between January 2019 and March 2025.

Inclusion criteria were as follows: (1) availability of preoperative abdominal ultrasound images obtained within 2 weeks before surgery; (2) postoperative pathological confirmation of PDAC; and (3) definitive LNM status determined by histopathological examination. Exclusion criteria included: (1) poor-quality or incomplete ultrasound images with severe artifacts or unclear tumor boundaries; (2) missing or incomplete clinicopathological data; (3) history of neoadjuvant chemotherapy or radiotherapy; or (4) coexistence of other malignancies.

After applying these criteria, a total of 99 patients were included in the final cohort, comprising 55 patients with LNM negative and 44 patients with LNM positive. All cases were randomly divided into a training cohort (*n* = 69, 70%) and a validation cohort (*n* = 30, 30%), maintaining a comparable LNM distribution between groups.

Clinical variables, including age, body mass index (BMI), tumor size, tumor location, and alpha-fetoprotein (AFP) levels, were collected from electronic medical records. Baseline characteristics were statistically compared between groups to confirm data balance.

This study was approved by the Ethics Committee of Shaoxing People’s Hospital. Given the retrospective design, the requirement for informed consent was waived, and all patient data were anonymized before analysis.

**Figure 1:**
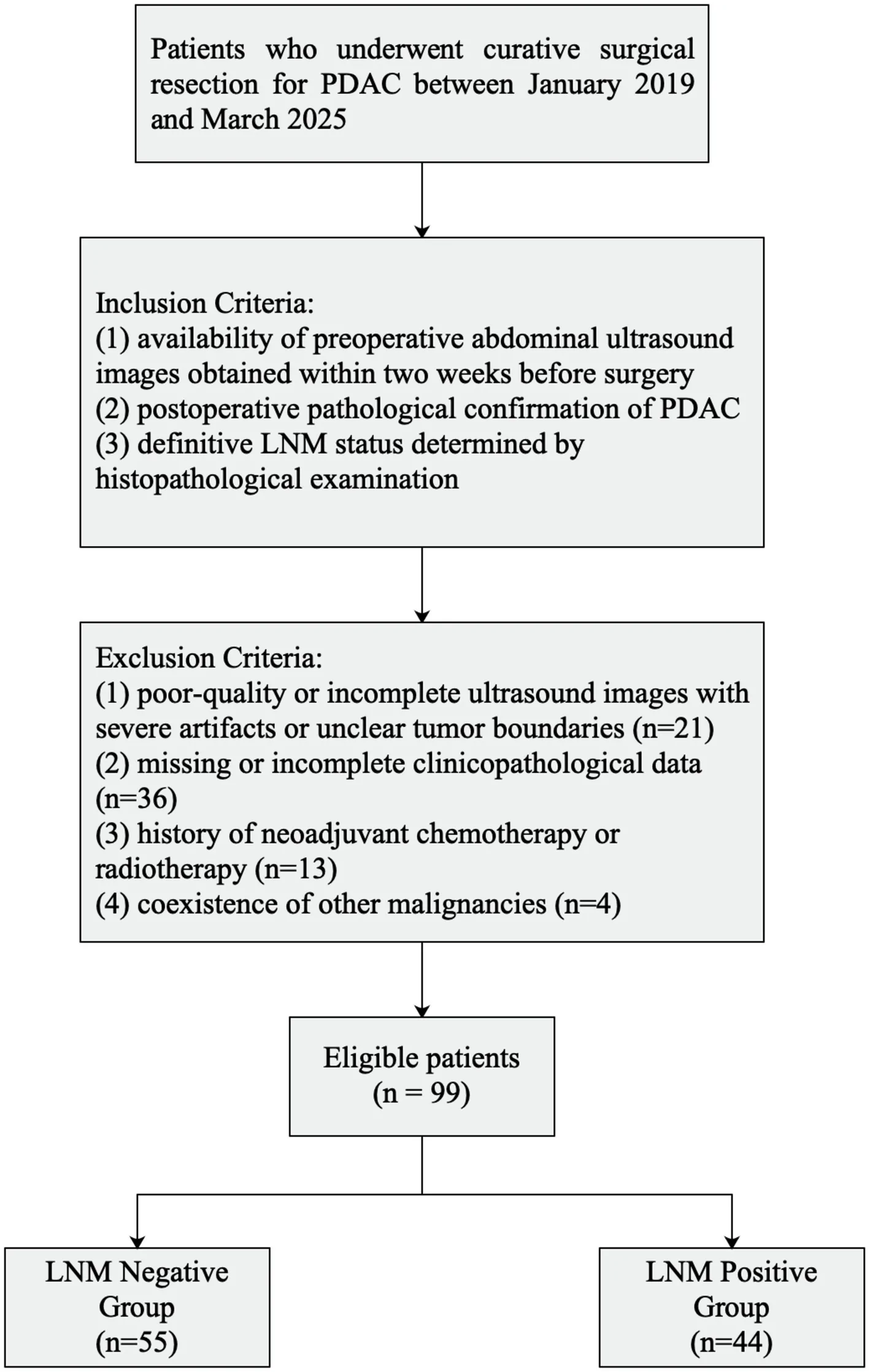
Patient selection flowchart.

The inclusion and exclusion workflow is illustrated in Figure 1.

### 2.2 Ultrasound acquisition and ROI segmentation

All preoperative abdominal ultrasound examinations were performed within 2 weeks before surgery using high-resolution ultrasound systems (specific device models and acquisition parameters are provided in the Supplementary materials). Each patient was examined in a supine position after overnight fasting to minimize bowel gas interference. Multiple grayscale images were obtained in different planes to ensure complete visualization of the pancreatic lesion, and the image showing the clearest tumor boundary was selected for analysis.

ROI delineation was performed using ITK-SNAP software (Version 4.4.0)^1^ (17). For each lesion, the intratumoral ROI was manually delineated along the visible tumor boundary to include the entire lesion. The peritumoral ROI was generated by automatically expanding the intratumoral contour outward by 3 mm using SimpleITK, followed by manual adjustment to exclude large vessels, bile ducts, and non-parenchymal regions (18). All ROIs were independently delineated by two ultrasound physicians (with 8 and 10 years of experience in abdominal ultrasound). Each physician repeated the delineation after an interval of one week to assess intra-observer and inter-observer consistency. Intraclass correlation coefficients (ICCs) were calculated for all cases, and features with ICC values greater than 0.75 were considered to have good reproducibility and retained for subsequent analysis.

### 2.3 Radiomics feature extraction and selection

Radiomics features were extracted using the open-source PyRadiomics package (version 3.0.1, Python 3.9). Before extraction, all ultrasound images and corresponding ROI masks were resampled to a uniform voxel size of 1.0 × 1.0 mm and discretized with a fixed bin width of 25 gray levels. For each ROI, a total of seven feature categories were extracted, including first-order statistics, shape descriptors, gray-level co-occurrence matrix (GLCM), gray-level run-length matrix (GLRLM), gray-level size zone matrix (GLSZM), gray-level dependence matrix (GLDM), and neighboring gray-tone difference matrix (NGTDM). In addition, wavelet and Laplacian of Gaussian (LoG) filters were applied to capture multi-scale textural information, resulting in a high-dimensional feature set for both intratumoral and peritumoral ROIs.

All extracted features were standardized using z-score normalization. Low-variance features were first removed, and then pairwise correlation analysis was applied to eliminate redundant features with a correlation coefficient greater than 0.90. After collinearity reduction, a two-sample t-test was performed to identify features that significantly differed between the LNM negative and LNM positive groups within the training cohort, and only features with *p* < 0.05 were retained. The remaining features were subsequently subjected to least absolute shrinkage and selection operator (LASSO) regression with 10-fold cross-validation to obtain the optimal subset of features. The selected features were used to construct independent radiomics signatures for the intratumoral and peritumoral ROIs.

### 2.4 Model construction

Separate predictive models were constructed based on intratumoral and peritumoral radiomics signatures. Model development was implemented using the scikit-learn framework (version 1.2.2, Python 3.9). To enable structured comparison rather than arbitrary trial-and-error selection, we evaluated a panel of representative machine learning algorithms covering diverse learning paradigms, including linear models (logistic regression), tree-based and ensemble methods (decision tree, random forest, gradient boosting, XGBoost, and AdaBoost), kernel-based methods (support vector machine), instance-based methods (k-nearest neighbor), and neural networks (multilayer perceptron), which are commonly applied in radiomics research. All models were trained exclusively within the training cohort, with hyperparameters optimized using cross-validation. Model performance was subsequently evaluated in an independent validation cohort that was not involved in any stage of model training or parameter tuning.

After establishing the individual intratumoral, peritumoral, and clinical models, several decision-level fusion strategies, including mean averaging, weighted averaging, rank-average fusion, the maximum probability rule, and logistic regression–based fusion, were evaluated within the training cohort. For the weighted averaging strategy, candidate weight combinations were evaluated in the training cohort. The optimal weights were then fixed and applied unchanged to the validation cohort.

For the logistic regression–based fusion model, the predicted probabilities from the intratumoral and peritumoral submodels were used as input variables, and fusion weights were derived solely within the training cohort. The estimated coefficients were then fixed and applied unchanged to the validation cohort for independent performance assessment, ensuring that no information from the validation data was used during weight estimation.

Hyperparameter tuning for individual submodels was conducted using a two-step strategy combining randomized search and grid search within a five-fold cross-validation framework, with the goal of reducing overfitting. The finalized models were subsequently applied to the validation cohort for independent evaluation.

### 2.5 Model evaluation

The diagnostic performance of each model was assessed in both the training and validation cohorts. Receiver operating characteristic (ROC) curves were generated, and the area under the curve (AUC) with corresponding 95% confidence intervals (CIs) was calculated to quantify discrimination ability. Differences in AUCs among models were compared using the DeLong test, a nonparametric method for evaluating correlated ROC curves (19). To further evaluate classification performance, confusion matrices were constructed to compute true positives (TP), false positives (FP), true negatives (TN), and false negatives (FN), from which sensitivity (TP/[TP + FN]), specificity (TN/[TN + FP]), accuracy ([TP + TN]/total), positive predictive value (PPV, TP/[TP + FP]), and negative predictive value (NPV, TN/[TN + FN]) were derived.

Model calibration was assessed using calibration curves and the Hosmer–Lemeshow goodness-of-fit test to determine the agreement between predicted and observed probabilities (20). Decision curve analysis (DCA) was performed to estimate the net clinical benefit of each model across a range of threshold probabilities (21).

These evaluation metrics were selected to provide a multi-dimensional assessment of model performance, encompassing discrimination, calibration, and clinical utility, rather than relying on a single performance indicator.

To examine the relationship between the intratumoral and peritumoral prediction results, Pearson and Spearman correlation analyses were conducted using their predicted probabilities. Bland–Altman plots were further generated to visualize the level of agreement between the two models.

Model interpretability was analyzed using the SHapley Additive exPlanations (SHAP) framework, a game-theory-based method that quantifies each feature’s marginal contribution to individual predictions (22). Global feature importance rankings and beeswarm plots were generated to illustrate the contribution of individual features to model predictions, enabling better understanding of the biological and clinical relevance of the radiomics features.

### 2.6 Statistical analysis

All statistical analyses were performed using Python (version 3.9) and R software (version 4.3.1). Continuous variables were expressed as mean ± standard deviation (SD) and compared between groups using the independent-sample t-test or Mann–Whitney U test, as appropriate. Categorical variables were compared using the chi-square test or Fisher’s exact test. Model performance was evaluated based on the area under the ROC curve (AUC), and differences in AUCs between models were compared using the DeLong test. Calibration performance was assessed using the Hosmer–Lemeshow test, and decision curve analysis (DCA) was used to quantify the net clinical benefit across varying threshold probabilities. Correlation between the prediction probabilities of intratumoral and peritumoral models was analyzed using Pearson and Spearman correlation coefficients, while agreement was further assessed using Bland–Altman analysis. A two-tailed *p* value < 0.05 was considered statistically significant.

## 3 Results

### 3.1 Characteristics of the study population

A total of 99 patients with pathologically confirmed PDAC were enrolled, including 55 with LNM negative and 44 with LNM positive. The detailed clinical characteristics are summarized in Table 1.

**Table 1:** Comparison of baseline characteristics between patients with and without lymph node metastasis in the overall, training, and validation cohorts.

Variables	LNM negative (*n* = 55)	LNM positive (*n* = 44)	*p*	Training cohort (*n* = 69)		*p*	Validation cohort (*n* = 30)		*p*
				LNM negative (*n* = 37)	LNM positive (*n* = 32)		LNM negative (*n* = 18)	LNM positive (*n* = 12)	
Age (year)	67.25 ± 7.71	67.68 ± 7.81	0.788	66.76 ± 8.25	67.0 ± 7.14	0.899	69.11 ± 5.75	69.67 ± 8.98	0.843
Tumor Size	3.55 ± 1.5	3.37 ± 1.09	0.494	3.67 ± 1.58	3.29 ± 1.08	0.255	3.31 ± 1.29	3.33 ± 1.17	0.968
BMI	22.36 ± 2.84	22.19 ± 2.21	0.752	22.42 ± 2.8	22.25 ± 2.45	0.786	22.57 ± 2.98	21.57 ± 1.87	0.328
AFP (ng/mL)	2.62 ± 1.15	2.97 ± 2.15	0.307	2.61 ± 1.04	2.68 ± 1.5	0.815	2.59 ± 1.36	3.62 ± 3.23	0.259
CEA (ng/mL)	5.44 ± 6.06	4.43 ± 2.77	0.316	6.04 ± 6.59	4.23 ± 2.72	0.159	4.27 ± 4.53	4.46 ± 2.18	0.9
CA125 (U/mL)	21.01 ± 16.33	24.33 ± 19.95	0.37	21.29 ± 18.16	20.83 ± 12.62	0.906	20.05 ± 11.82	29.19 ± 29.19	0.26
CA199 (U/mL)	1021.38 ± 2812.42	1859.34 ± 2676.42	0.14	928.59 ± 2676.69	1569.4 ± 2628.94	0.328	1208.93 ± 3064.98	2530.22 ± 2717.9	0.252
ALT (IU/L)	76.09 ± 119.23	100.99 ± 120.93	0.312	100.61 ± 140.63	102.59 ± 126.19	0.952	17.9 (12.65–46.25)	50.15 (13.65–180.15)	0.074
AST (IU/L)	50.51 ± 63.39	80.43 ± 116.44	0.11	61.05 ± 74.59	86.95 ± 131.34	0.317	26.05 (19.3–36.2)	44.4 (18.5–120.75)	0.065
ALB (g/L)	39.47 ± 4.58	38.14 ± 4.2	0.145	39.02 ± 4.65	38.34 ± 4.24	0.537	40.23 ± 4.34	37.3 ± 4.0	0.082
TBIL (μmol/L)	58.64 ± 99.39	82.94 ± 95.72	0.227	74.14 ± 115.95	82.6 ± 94.76	0.747	13.7 (10.15–21.9)	45.15 (12.35–190.05)	0.096
DBIL (μmol/L)	33.92 ± 65.56	50.12 ± 64.63	0.227	43.78 ± 75.74	53.25 ± 67.0	0.592	22.02 ± 42.33	52.57 ± 61.16	0.13
PT (s)	12.47 ± 1.38	12.76 ± 4.1	0.627	12.61 ± 1.51	12.97 ± 4.76	0.666	12.17 ± 0.99	12.21 ± 0.83	0.92
INR	0.99 ± 0.08	0.98 ± 0.09	0.405	0.99 ± 0.08	0.98 ± 0.09	0.665	0.99 ± 0.07	0.97 ± 0.09	0.641
Gender			0.785			0.883			0.626
Female	24	18		18	15		6	3	
Male	31	26		19	17		12	9	
Smoking			0.914			0.986			0.745
No	43	34		30	26		13	8	
Yes	12	10		7	6		5	4	
Diabetes			0.177			0.084			0.866
No	40	37		26	28		13	9	
Yes	15	7		11	4		5	3	
Tumor location			<0.001^*^			0.012^*^			0.004^*^
Head	24	35		18	25		7	11	
Body or Tail	31	9		19	7		11	1	
Drinking alcohol			0.6			0.553			0.86
No	46	35		32	26		14	9	
Yes	9	9		5	6		4	3	
Hypertension			0.719			0.762			0.232
No	28	24		21	17		8	8	
Yes	27	20		16	15		10	4	

Continuous variables are presented as mean ± standard deviation or median (interquartile range), as appropriate; categorical variables are presented as numbers. BMI, body mass index; AFP, alpha-fetoprotein; CEA, carcinoembryonic antigen; CA125, cancer antigen 125; CA199, carbohydrate antigen 19–9; ALT, alanine aminotransferase; AST, aspartate aminotransferase; ALB, albumin; TBIL, total bilirubin; DBIL, direct bilirubin; PT, prothrombin time; INR, international normalized ratio. An asterisk indicates *p* < 0.05.

Between the LNM negative and LNM positive groups, only tumor location showed a statistically significant difference (*p* < 0.05), with LNM positive tumors more frequently located in the pancreatic head. Other variables, including age, tumor size, BMI, and AFP levels, did not differ significantly between the two groups (all *p* > 0.05).

When comparing the training cohort (*n* = 69) and the validation cohort (*n* = 30), there were no significant differences in any clinical characteristics (all *p* > 0.05), confirming that the dataset was evenly distributed (Supplementary Table S1).

### 3.2 Feature selection and reproducibility

A total of 1,495 radiomics features were initially extracted from each of the intratumoral and peritumoral ROIs, encompassing first-order, shape, and texture features, together with wavelet- and LoG-filtered transformations.

After intra- and inter-observer reproducibility assessment across all cases, 1,402 features from the intratumoral ROI and 1,399 features from the peritumoral ROI demonstrated good repeatability, with ICCs greater than 0.75. Only features with ICC ≤ 0.75 were excluded from further analysis.

The retained features were standardized using z-score normalization. Features with low variance were first removed, and highly correlated features—defined as those with an absolute correlation coefficient greater than 0.90—were eliminated to reduce collinearity. Subsequently, two-sample t-tests were performed to identify features that significantly differed between the LNM negative and LNM positive groups, and features with *p* < 0.05 were retained. The remaining features were then refined using LASSO regression with 10-fold cross-validation, where the optimal penalty parameter (*λ*) corresponding to the minimum cross-validated error was selected to obtain the final subset of features (Supplementary Figures S1, S2).

The selected intratumoral and peritumoral features were used to construct their respective radiomics signatures, which served as the input for model construction. Detailed feature lists and coefficients are provided in Table 2.

**Table 2:** Radiomic features selected by LASSO regression and their coefficients in the intratumoral and peritumoral models.

Model	Filter	Feature class	Feature	Coefficient
Intratumoral	wavelet-LHL	GLDM	Dependence Non Uniformity Normalized	−0.002702
	wavelet-HLL	GLCM	IDMN	0.080677
Peritumoral	wavelet-HHL	First-order	Minimum	−0.070828
	wavelet-HHH	First-order	Kurtosis	0.005634

### 3.3 Model performance

Nine machine learning algorithms, including support vector machine (SVM), random forest (RF), k-nearest neighbor (KNN), logistic regression (LR), decision tree (DT), multilayer perceptron (MLP), AdaBoost, gradient boosting (GB), and extreme gradient boosting (XGBoost), were evaluated for model construction based on both intratumoral and peritumoral radiomics signatures.

**Figure 2:**
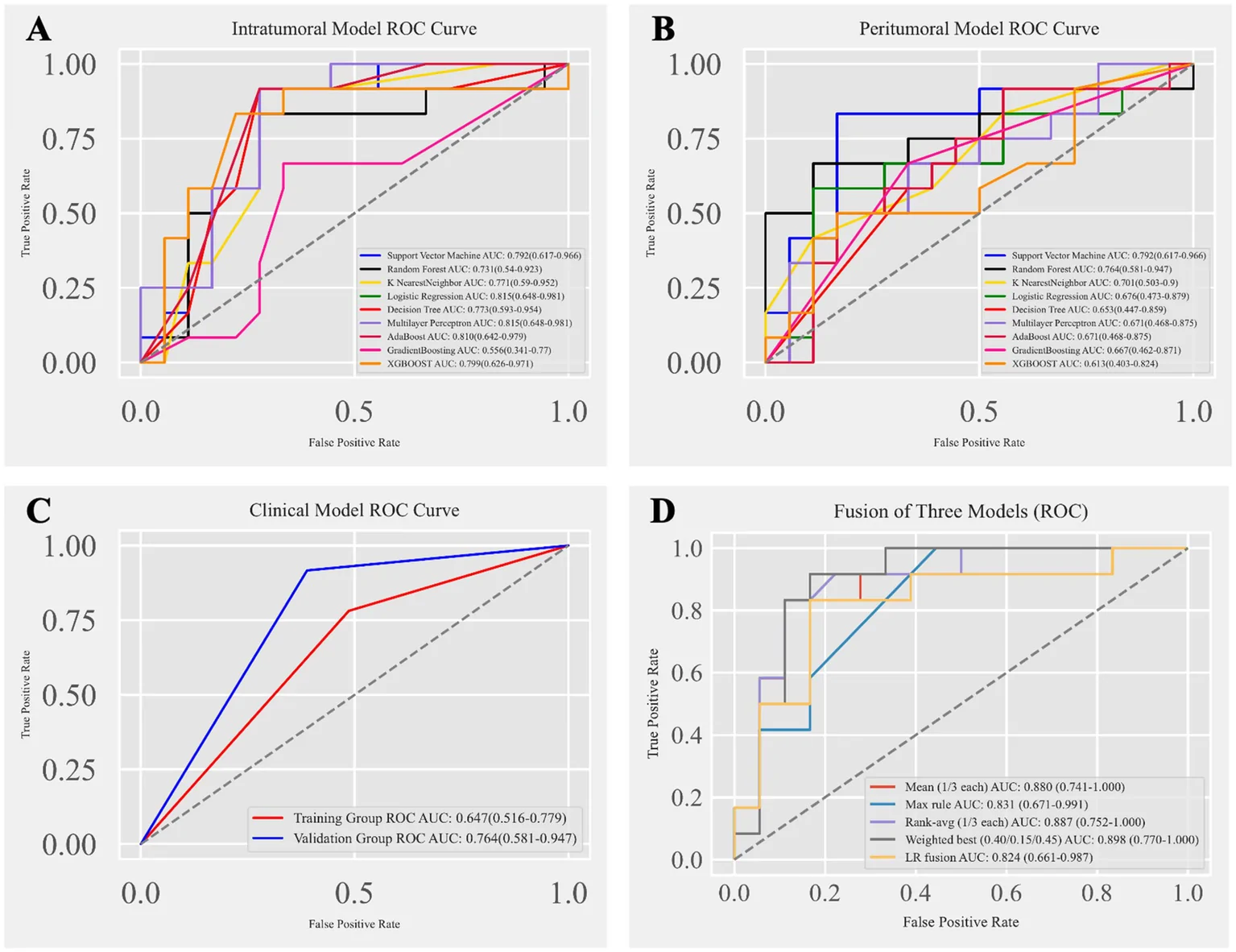
ROC curves of different modeling strategies for predicting lymph node metastasis in pancreatic ductal adenocarcinoma. **(A)** ROC curves of nine machine learning algorithms based on the intratumoral radiomics signature, with the logistic regression model achieving the highest AUC (0.815). **(B)** ROC curves of different algorithms for the peritumoral signature, where the random forest model performed best (AUC = 0.792). **(C)** ROC curves of the clinical model constructed using preoperative variables, including tumor location (head vs. body/tail), showing moderate discrimination in both training and validation cohorts. **(D)** ROC curves of different fusion strategies combining the intratumoral, peritumoral, and clinical models. The weighted fusion model (0.40 × intratumoral + 0.15 × peritumoral + 0.45 × clinical) achieved the highest AUC (0.898) and was selected as the final combined model. ROC, receiver operating characteristic; AUC, area under the curve.

As shown in Figures 2A,B, most algorithms achieved favorable discrimination in predicting lymph node metastasis. The logistic regression model achieved the best performance for the intratumoral signature (AUC = 0.815, 95% CI: 0.648–0.981), while the random forest model performed best for the peritumoral signature (AUC = 0.792, 95% CI: 0.617–0.966). For clinical reference, a model incorporating significant preoperative clinical variables—including tumor location (head vs. body/tail)—was developed, achieving moderate predictive performance in both the training and validation cohorts (AUC = 0.647 and 0.764, respectively; Figure 2C).

Subsequently, the prediction probabilities from the intratumoral, peritumoral, and clinical models were integrated using multiple fusion strategies, including mean averaging, weighted averaging, rank-average fusion, maximum probability rule, and logistic regression–based fusion. As shown in Figure 2D, different fusion strategies yielded varying diagnostic performance, with the weighted fusion model (0.40 × intratumoral + 0.15 × peritumoral + 0.45 × clinical) demonstrating an AUC of 0.898 (95% CI: 0.770–1.000). The optimal weights for the weighted fusion model were determined within the training cohort and were subsequently fixed and applied unchanged to the validation cohort without further adjustment to avoid information leakage. This model was carried forward as the combined model in subsequent analyses.

Pairwise DeLong comparisons showed no statistically significant differences among the validation-cohort AUCs of the four models (all *p* > 0.05; Supplementary Table S2). The diagnostic metrics of each model—including sensitivity, specificity, accuracy, PPV, and NPV—are summarized in Table 3. These results indicate that the weighted combined model showed numerically higher overall metrics than the single-source models.

**Table 3:** Diagnostic performance metrics of the intratumoral, peritumoral, clinical, and combined models.

Model	TP	FP	TN	FN	AUC (95% CI)	Sensitivity	Specificity	Precision	NPV	Accuracy	F1
Intratumoral	11	5	13	1	0.815 (0.648–0.981)	0.917	0.722	0.687	0.929	0.8	0.786
Peritumoral	10	3	15	2	0.792 (0.617–0.966)	0.833	0.833	0.769	0.882	0.833	0.8
Clinical	11	7	11	1	0.764 (0.581–0.947)	0.917	0.611	0.611	0.917	0.733	0.733
Combined	11	3	15	1	0.898 (0.770–1.000)	0.917	0.833	0.786	0.937	0.867	0.846

TP, true positive; FP, false positive; TN, true negative; FN, false negative; AUC, area under the curve; NPV, negative predictive value; CI, confidence interval.

### 3.4 Comparison among models

**Figure 3:**
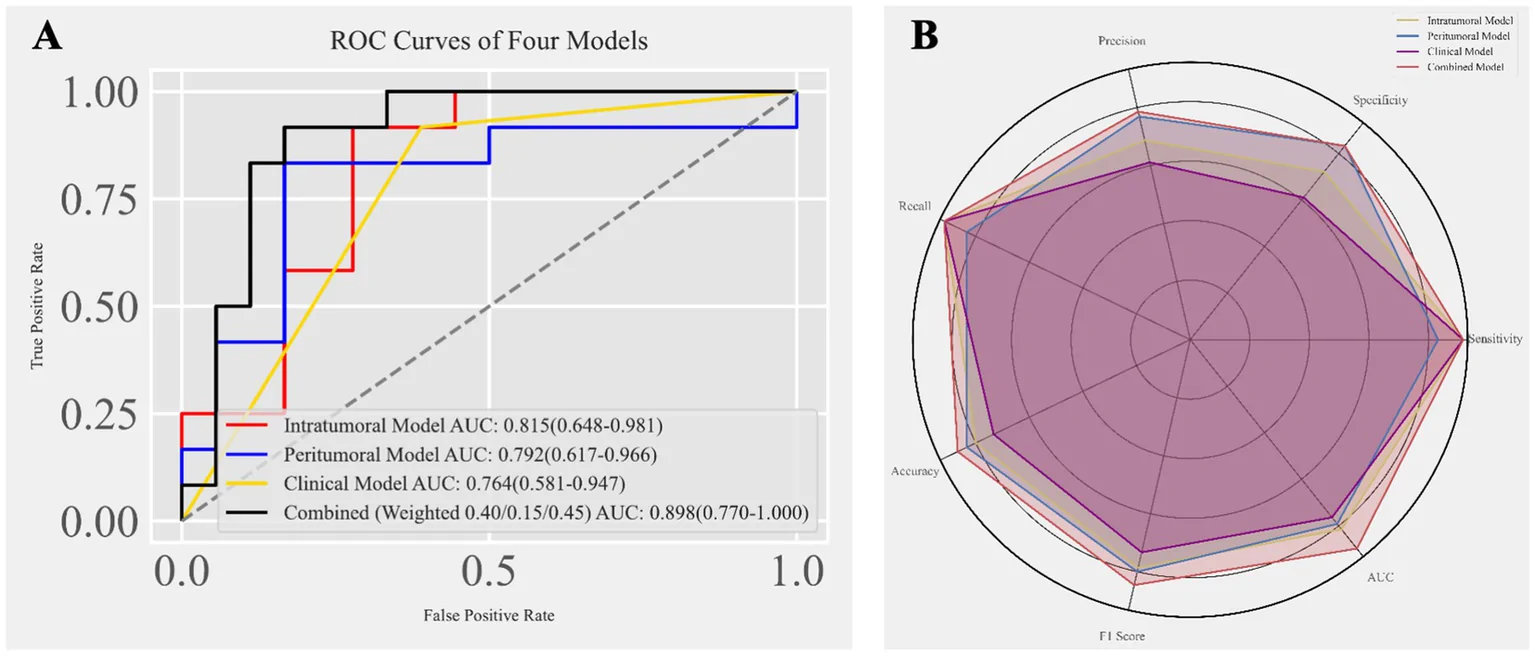
Comparative performance of four models for predicting lymph node metastasis in pancreatic ductal adenocarcinoma. **(A)** ROC curves of the intratumoral, peritumoral, clinical, and weighted combined models (0.40 × intratumoral + 0.15 × peritumoral + 0.45 × clinical). The combined model yielded the highest AUC (0.898, 95% CI: 0.770–1.000). **(B)** Radar chart summarizing multiple diagnostic indicators (AUC, sensitivity, specificity, precision, recall, accuracy, and F1-score), illustrating that the combined model provided the most stable and comprehensive predictive performance. ROC, receiver operating characteristic; AUC, area under the curve; CI, confidence interval.

**Figure 4:**
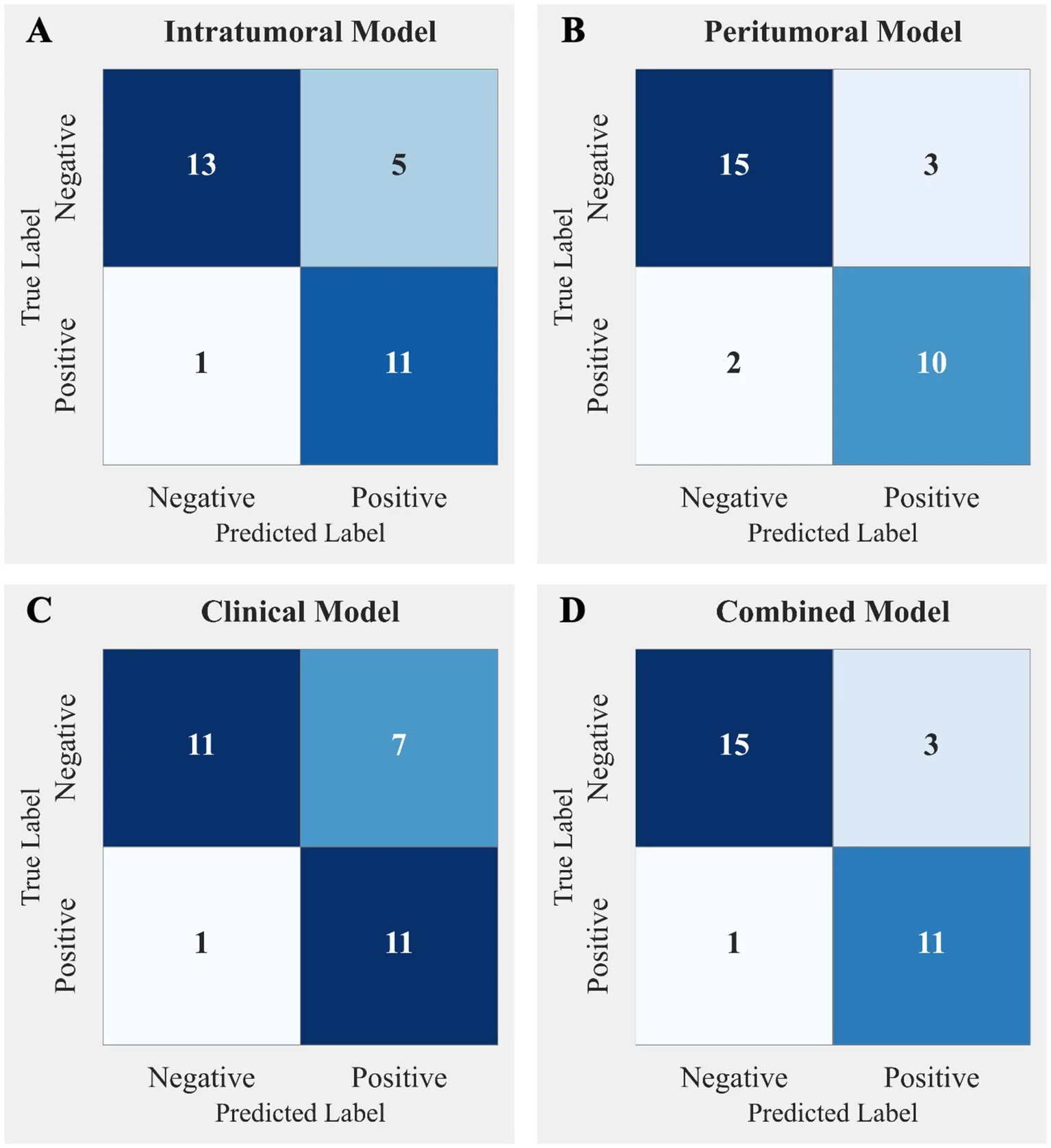
Confusion matrices of the four models for predicting lymph node metastasis. **(A)** Intratumoral model, **(B)** Peritumoral model, **(C)** Clinical model, and **(D)** Combined model (weighted 0.40/0.15/0.45). The combined model achieved the highest overall accuracy with the fewest misclassifications among all models.

The overall diagnostic performance of the four final models—including the intratumoral, peritumoral, clinical, and weighted combined models—is summarized in Figure 3. The ROC curves (Figure 3A) demonstrate that the combined model achieved the best overall discrimination, followed by the intratumoral and peritumoral models. The radar chart (also shown in Figure 3B) illustrates the distribution of multiple performance metrics (AUC, sensitivity, specificity, precision, recall, accuracy, and F1-score) across the evaluated models. The combined model exhibited a relatively larger coverage across these metrics, reflecting a more balanced performance profile within this cohort. The confusion matrices (Figure 4) further visualize the prediction distribution across positive and negative cases, where the combined model achieved the highest number of correctly classified samples in both categories.

### 3.5 Calibration and decision curve analysis

**Figure 5:**
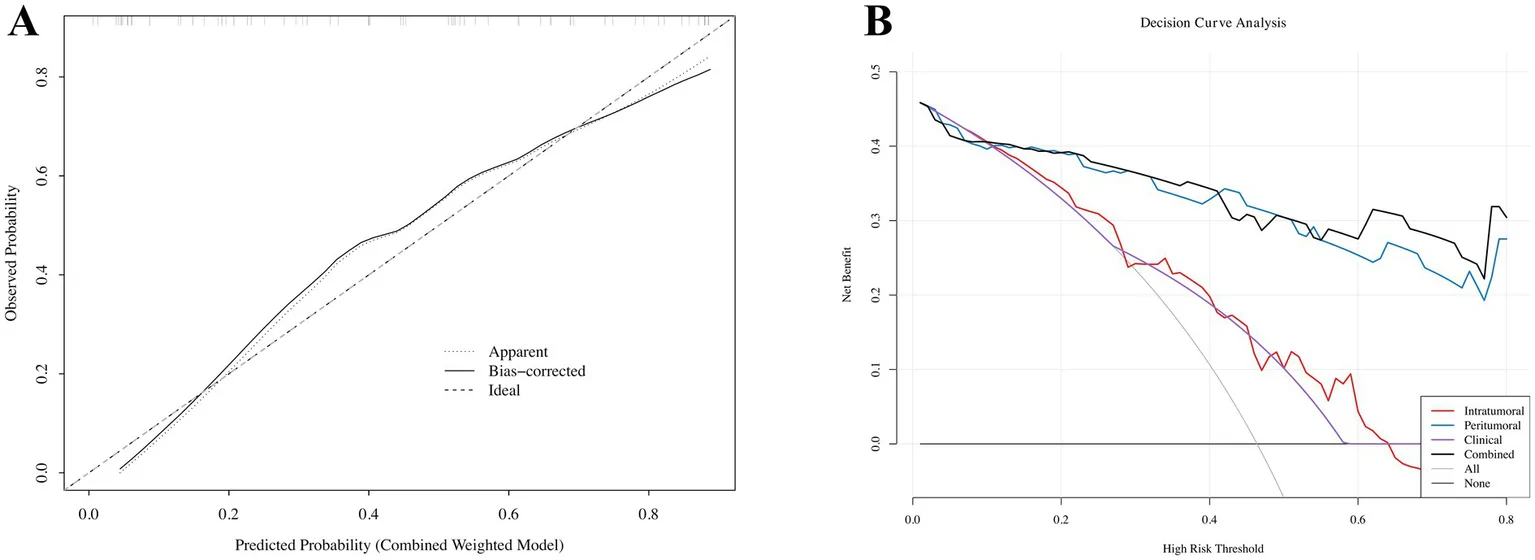
Calibration and decision curve analysis of the combined model. **(A)** Calibration curve of the combined weighted model (0.40 × intratumoral + 0.15 × peritumoral + 0.45 × clinical) showing good agreement between predicted and observed probabilities of lymph node metastasis. **(B)** Decision curve analysis comparing the net clinical benefit of the intratumoral, peritumoral, clinical, and combined models. The combined model provided the highest net benefit across most threshold probabilities.

The calibration performance of the weighted combined model is shown in Figure 5A, demonstrating good agreement between predicted and observed probabilities in the validation cohort. The mean absolute error (MAE) and mean squared error (MSE) were 0.04 and 0.00209, respectively, indicating minimal deviation between predicted and actual outcomes. The Hosmer–Lemeshow goodness-of-fit test yielded χ^2^ = 13.658, df = 8, *p* = 0.091, suggesting no significant difference between predicted and observed values and confirming good model calibration.

As illustrated in the decision curve analysis (Figure 5B), the combined model showed higher net benefit across a wide range of threshold probabilities relative to the other models. These results indicate that the weighted combined model not only achieves good calibration but also offers greater potential clinical benefit for individualized prediction of lymph node metastasis in PDAC.

### 3.6 Model interpretability and inter-model relationship analysis

**Figure 6:**
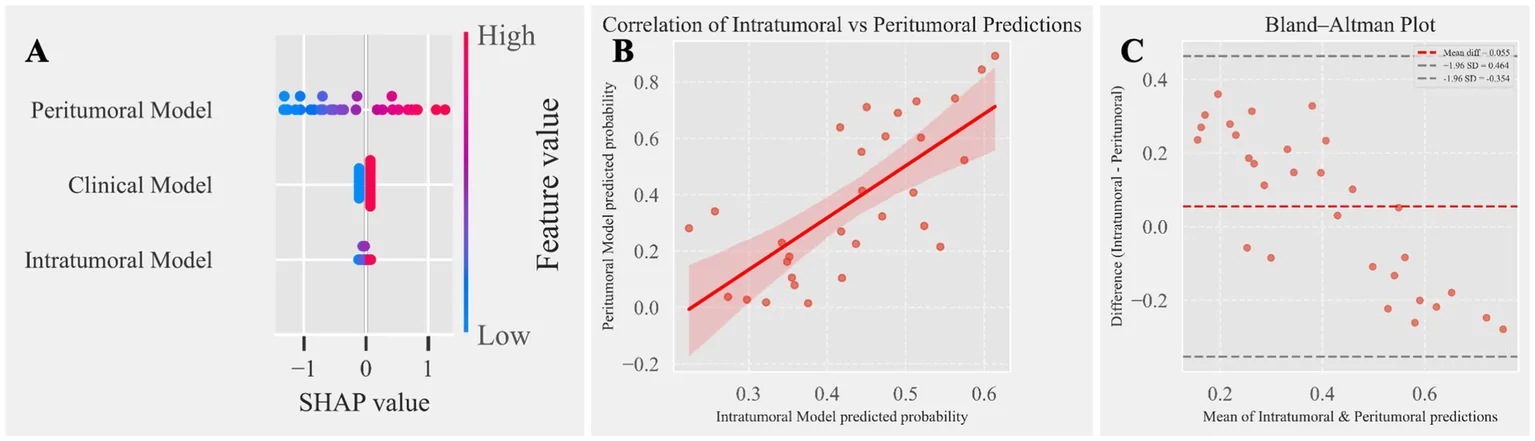
Interpretability and correlation analysis of the combined model. **(A)** SHAP summary plot showing the relative contributions of the intratumoral, peritumoral, and clinical models to the final combined prediction, with the peritumoral model having the greatest influence. **(B)** Scatter plot demonstrating a significant positive correlation between intratumoral and peritumoral model predictions (Pearson *r* = 0.711, *p* < 0.001). **(C)** Bland–Altman plot illustrating the distribution of differences between intratumoral and peritumoral predictions, with the mean difference and 95% limits of agreement shown. SHAP, Shapley Additive Explanations.

Model interpretability was evaluated using SHAP analysis applied to the decision-level combined model. As shown in Figure 6A, the SHAP beeswarm plot demonstrated that, at the individual-patient level, the peritumoral model output contributed more prominently to the final prediction in a subset of cases, whereas the intratumoral and clinical model outputs showed variable contributions across patients. Notably, the relative importance indicated by SHAP values was not strictly aligned with the predefined fusion weights assigned to the intratumoral, peritumoral, and clinical models in the combined model. This discrepancy arises because SHAP values reflect local, case-specific contributions of each submodel to individual predictions, whereas the fusion weights represent global coefficients that summarize the average influence of each submodel across the entire cohort during model construction. Therefore, differences between SHAP-derived contribution patterns and fusion weight magnitudes reflect heterogeneity in patient-specific prediction mechanisms rather than inconsistency in the modeling strategy.

To explore this relationship, correlation and consistency analyses were conducted between the intratumoral and peritumoral prediction probabilities. As illustrated in Figures 6B, a strong positive correlation was observed (Pearson’s *r* = 0.711, *p* = 1.07 × 10^−5^; Spearman’s *r* = 0.693, *p* = 2.19 × 10^−5^), indicating that the two models showed a high degree of association in their prediction patterns.

The Bland–Altman plot (Figure 6C) illustrated the mean difference and 95% limits of agreement between the intratumoral and peritumoral predictions, with most points lying within these limits. Importantly, a few cases exhibited noticeable deviations, suggesting that while the intratumoral and peritumoral models were generally concordant, they also provided complementary information by emphasizing different image-derived phenotypes. This combination of global agreement and local complementarity supports the rationale for integrating both regions in the final fusion model.

## 4 Discussion

Our study developed and compared ultrasound-based intratumoral, peritumoral, clinical, and combined radiomics models for the preoperative prediction of LNM in patients with PDAC. Both intratumoral and peritumoral radiomics models demonstrated moderate-to-good discriminatory performance within this cohort. The combined model integrating multi-regional radiomics signatures yielded an AUC of 0.898. While no statistically significant differences were observed among models according to the DeLong test, the combined model showed a more balanced sensitivity–specificity profile, along with favorable calibration characteristics and higher net benefit across a range of threshold probabilities in decision curve analysis. Collectively, these findings indicate that ultrasound-based radiomics features capture complementary information beyond conventional clinical parameters, and that integrating intratumoral and peritumoral imaging information may facilitate more comprehensive characterization of PDAC-related nodal status.

Beyond discrimination performance, model interpretability and inter-model relationship analyses provided deeper insights into the distinct yet complementary roles of intratumoral and peritumoral regions. The SHAP analysis revealed that peritumoral features contributed more substantially to the final combined prediction, implying that the peritumoral zone—representing the tumor–microenvironment interface—plays a critical role in the metastatic process. Inter-model relationship analysis reflected the degree of association between the two single-region models, while Bland–Altman analysis was used to visualize the distribution of differences across their predicted probabilities. Together, these findings suggest that while the two models capture overlapping information, they also provide region-specific complementary insights, emphasizing different but synergistic imaging phenotypes relevant to LNM prediction.

From a biological standpoint, the complementary value of intratumoral and peritumoral regions can be explained by their distinct pathophysiological implications (23). Intratumoral features primarily characterize the internal heterogeneity of PDAC, such as necrosis, cellular density, and vascular irregularity, which are closely associated with tumor grade and aggressiveness (24). In contrast, peritumoral features capture subtle changes in the adjacent stroma, including inflammatory cell infiltration, fibroblastic activation, and early lymphovascular invasion (25). The integration of these two imaging domains thus provides a more comprehensive depiction of tumor biology, aligning with the multi-compartmental nature of PDAC progression and its propensity for lymphatic dissemination (26).

In addition to biological complementarity, the present study also highlights the methodological value of decision-level fusion. Unlike feature-level integration, which can introduce redundancy and overfitting due to the high dimensionality of radiomics data, decision-level fusion combines the predictive probabilities of independently trained models (27). This strategy preserves the interpretability of each submodel while allowing complementary information to emerge at the decision level, yielding a more stable and clinically feasible integration framework (28). The consistent, though statistically nonsignificant, increase in AUC observed in our study supports the concept that model fusion should prioritize model reliability and interpretability over purely numerical gains.

Interestingly, the SHAP-derived feature contributions did not fully align with the assigned fusion weights. This discrepancy suggests that the relative contribution of each region may vary dynamically among patients, influenced by intertumoral heterogeneity and local microenvironmental conditions (29). In other words, the diagnostic importance of intratumoral versus peritumoral information is context-dependent—some cases rely more on internal tumor heterogeneity, whereas others depend on stromal and peritumoral changes. This observation underscores the importance of interpretability analysis in radiomics, as understanding model behavior should extend beyond static coefficients to capture individualized imaging–biology relationships.

Previous radiomics studies based primarily on CT or MRI have validated the diagnostic and prognostic value of peritumoral features in various malignancies, including hepatocellular carcinoma, intrahepatic cholangiocarcinoma, and breast cancer (15, 30, 31). However, ultrasound-based peritumoral analysis remains limited, especially in PDAC. The present findings extend this concept to ultrasound imaging and demonstrate that peritumoral radiomics features extracted from routine B-mode images can provide nonredundant information for LNM prediction.

From a clinical perspective, accurate preoperative assessment of LNM is essential for determining surgical strategies, including the extent of lymphadenectomy and the selection of candidates for neoadjuvant therapy (32). In this study, the radiomics-based models showed numerically higher performance than the clinical model within this cohort, suggesting that quantitative ultrasound features capture microstructural and microenvironmental characteristics not reflected by traditional clinical variables. The proposed combined model, built upon widely available ultrasound images, offers a noninvasive and interpretable tool for individualized risk stratification. Given the real-time, cost-effective, and radiation-free nature of ultrasound, this framework has strong potential for integration into routine preoperative assessment or intraoperative decision support, thereby contributing to precision surgery in PDAC. In practical terms, patients predicted to be at high risk of LNM may benefit from more comprehensive lymph node evaluation, intensified perioperative monitoring, and multidisciplinary discussion regarding systemic treatment strategies. Conversely, low-risk patients may potentially be managed with more individualized surgical planning. Importantly, we emphasize that this model is intended to complement—rather than replace—standard clinical and pathological assessment.

Several limitations should be acknowledged. First, this was a single-center retrospective study with a relatively small sample size, which may limit the generalizability of the findings. Second, although manual ROI delineation demonstrated good reproducibility, it remains operator-dependent. Third, only grayscale ultrasound images were analyzed; future research should explore multimodal imaging data, such as contrast-enhanced ultrasound or elastography, to capture complementary information. Moreover, combining radiomics features with clinical data or deep learning–based image representations could further enhance predictive performance and external validity.

## 5 Conclusion

In conclusion, this study demonstrated that ultrasound-based intratumoral and peritumoral radiomics features, when integrated with clinical information, provide complementary insights into the biological and structural characteristics of PDAC. The combined model achieved numerically higher overall performance with favorable calibration within this cohort. These results highlight the potential of multi-regional ultrasound radiomics as an interpretable imaging-based approach for preoperative risk stratification in PDAC, meriting further investigation in larger and independent cohorts.

## Data Availability

The original contributions presented in the study are included in the article/Supplementary material, further inquiries can be directed to the corresponding author.

## Footnotes

1
www.itksnap.org

## Glossary

PDAC: Pancreatic ductal adenocarcinoma
LNM: Lymph node metastasis
CT: Computed tomography
MRI: Magnetic resonance imaging
PET: Positron emission tomography
US: Ultrasound
ROI: Region of interest
ICC: Intraclass correlation coefficient
GLCM: Gray-level co-occurrence matrix
GLRLM: Gray-level run-length matrix
GLSZM: Gray-level size zone matrix
GLDM: Gray-level dependence matrix
NGTDM: Neighboring gray-tone difference matrix
LoG: Laplacian of Gaussian
LASSO: Least absolute shrinkage and selection operator
SVM: Support vector machine
RF: Random forest
KNN: k-nearest neighbor
LR: Logistic regression
DT: Decision tree
MLP: Multilayer perceptron
GB: Gradient boosting
XGBoost: Extreme gradient boosting
ROC: Receiver operating characteristic
AUC: Area under the curve
CI: Confidence interval
TP: True positive
FP: False positive
TN: True negative
FN: False negative
PPV: Positive predictive value
NPV: Negative predictive value
DCA: Decision curve analysis
SHAP: SHapley Additive exPlanations
MAE: Mean absolute error
MSE: Mean squared error
SD: Standard deviation
