# Supplementary material for: Ultrasound-based intratumoral and peritumoral radiomics for preoperative prediction of lymph node metastasis in pancreatic ductal adenocarcinoma
Source: Front Med (Lausanne). 2026 Aug 19;13:1793048. doi: 10.3389/fmed.2026.1793048 (PMC13533701; doi:10.3389/fmed.2026.1793048)
Supplement: Supplementary file 1 [file Data_Sheet_1.pdf]

i. Ultrasound machines

Ultrasound examination was performed by using one of the following ultrasound machines: LOGIQ E8 (GE Healthcare, United States; C5-1 convex array probes, 1–5 MHz); LOGIQ E9 (GE Healthcare, United States; C5-1 convex array probes, 1–5 MHz); Aplio 500 (Toshiba Medical systems, Japan; 6C1 probe, 1–6 MHz); i800 (Cannon Medical systems Corporation, Japan; i8CX1 probe, 1-8MHz); and Resona 7T (Mindray, China; SC6-1 U probe, 1-6MHz).

Table S1. Comparison of clinical characteristics between the training and validation cohorts

Variables	Training cohort (n = 69)	Validation cohort (n = 30)	p
**Age (year)**	66.87 ± 7.76	69.33 ± 7.22	0.145
**Tumor Size**	3.49 ± 1.38	3.31 ± 1.24	0.543
**BMI**	22.34 ± 2.64	22.17 ± 2.64	0.772
**AFP (ng/mL)**	2.64 ± 1.27	3.0 ± 2.35	0.334
**CEA (ng/mL)**	5.2 ± 5.25	4.34 ± 3.77	0.427
**CA125 (U/mL)**	21.08 ± 15.84	23.71 ± 21.09	0.5
**CA199 (U/mL)**	1225.78 ± 2673.82	1737.44 ± 3001.71	0.406
**ALT (IU/L)**	101.53 ± 134.13	62.5 ± 77.62	0.144
**AST (IU/L)**	73.06 ± 105.59	47.1 ± 42.85	0.201
**ALB (g/L)**	38.7 ± 4.48	39.06 ± 4.45	0.72
**TBIL (µmol/L)**	78.06 ± 106.73	62.75 ± 82.7	0.49
**DBIL (µmol/L)**	48.17 ± 71.97	34.24 ± 52.87	0.347
**PT (s)**	12.78 ± 3.43	12.19 ± 0.93	0.361
**INR**	0.98 ± 0.08	0.98 ± 0.08	0.934
**Gender**			0.099
**Female**	33	9	
**Male**	36	21	
**Smoking**			0.22
**No**	56	21	
**Yes**	13	9	
**Diabetes**			0.594
**No**	54	22	
**Yes**	15	8	
**Tumor location**			0.827
**Head**	43	18	

**Body or Tail**	26	12	
**Drinking Alcohol**			0.381
**No**	58	23	
**Yes**	11	7	
**Hypertension**			0.873
**No**	38	16	
**Yes**	31	14	

BMI, body mass index; AFP, alpha-fetoprotein; CEA, carcinoembryonic antigen; CA125, cancer antigen 125; CA199, carbohydrate antigen 19-9; ALT, alanine aminotransferase; AST, aspartate aminotransferase; ALB, albumin; TBIL, total bilirubin; DBIL, direct bilirubin; PT, prothrombin time; INR, international normalized ratio; $p < 0.05$ indicates a statistically significant difference.

Table S2. Pairwise DeLong comparisons of validation-cohort AUCs.

**Model 1**	**Model 2**	**p value**
Intratumoral	Peritumoral	0.793
Intratumoral	Clinical	0.638
Intratumoral	Combined	0.211
Peritumoral	Clinical	0.817
Peritumoral	Combined	0.115
Clinical	Combined	0.063

AUC, area under the curve. All p values are two-sided.

Figure S1. Intratumoral model LASSO procedure

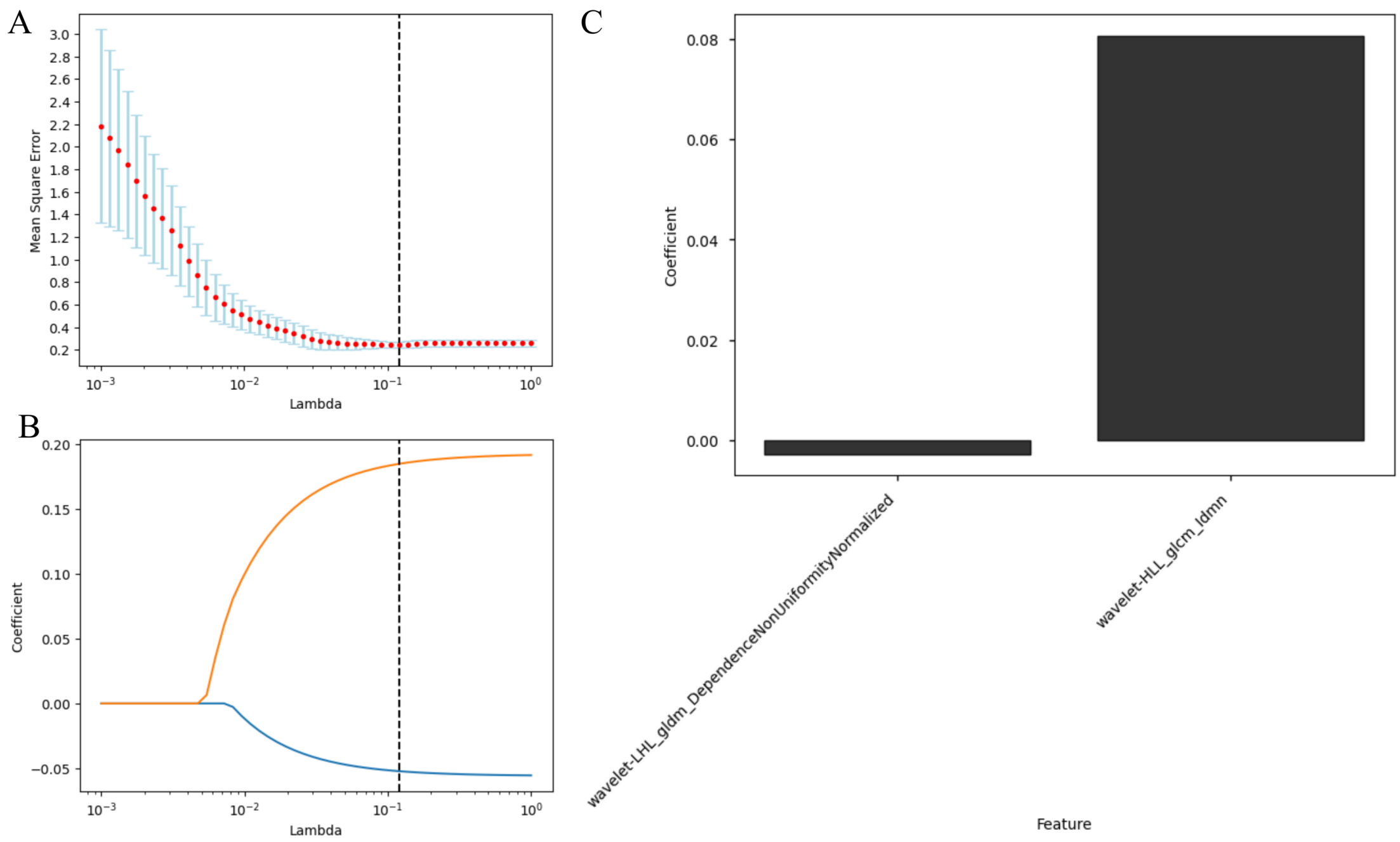

Figure S1. (A, B) Least absolute shrinkage and selection operator (LASSO) regression complexity was controlled using a tuning parameter lambda; the optimal lambda value for the minimized mean squared error was 0.1206792640639329. (C) The 2 selected variables and their coefficient values are shown.

Figure S2. Peritumoral model LASSO procedure

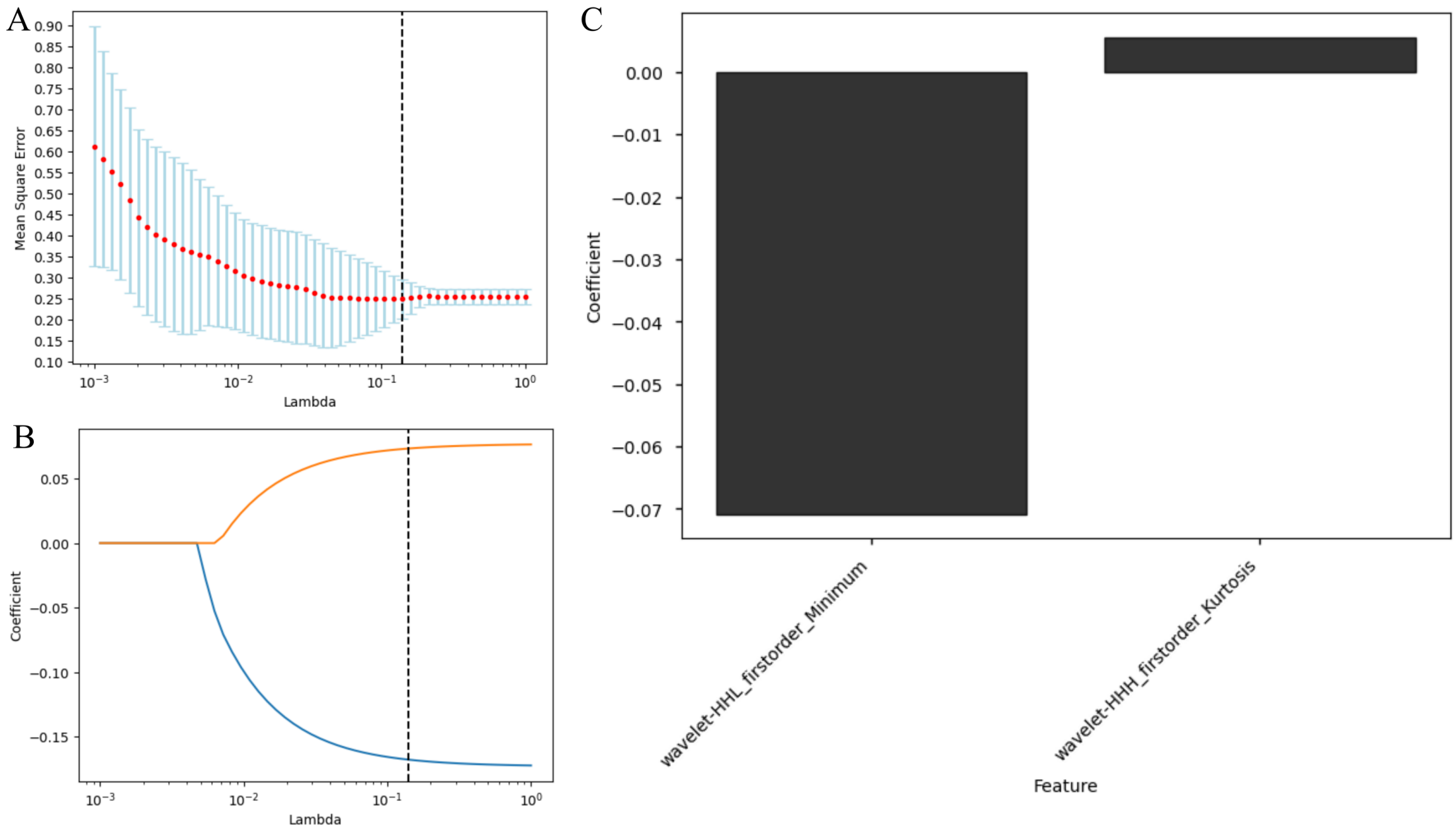

Figure S2. (A, B) Least absolute shrinkage and selection operator (LASSO) regression complexity was controlled using a tuning parameter lambda; the optimal lambda value for the minimized mean squared error was 0.13894954943731375. (C) The 2 selected variables and their coefficient values are shown.
